# Kinetics of small and middle molecule clearance during continuous hemodialysis

**DOI:** 10.1038/s41598-023-40075-y

**Published:** 2023-08-09

**Authors:** Livia Whiting, Nathan Bianchi, Mohamed Faouzi, Antoine Schneider

**Affiliations:** 1 Service de Médecine Intensive Adulte (SMIA), Centre Hospitalier Universitaire Vaudois (CHUV), Rue du Bugnon 46, 1011 Lausanne, Switzerland; 2 Division of Biostatistics, Center for Primary Care and Public Health (UNISANTE), University of Lausanne, Lausanne, Switzerland; 3 Faculty of Biology and Medicine, University of Lausanne, Lausanne, Switzerland

**Keywords:** Medical research, Nephrology

## Abstract

Regional citrate anticoagulation (RCA) enables prolonged continuous kidney replacement therapy (CKRT) filter lifespan. However, membrane diffusive performance might progressively decrease and remain unnoticed. We prospectively evaluated the kinetics of solute clearance and factors associated with decreased membrane performance in 135 consecutive CKRT-RCA circuits (35 patients). We recorded baseline patients’ characteristics and clinical signs of decreased membrane performance. We calculated effluent/serum ratios (ESR) as well as respective clearances for urea, creatinine and β2-microglobuline at 12, 24, 48 and 72 h after circuit initiation. Using mixed-effects logistic regression model analyses, we assessed the effect of time on those values and determined independent predictors of decreased membrane performance as defined by an ESR for urea < 0.81. We observed a minor but statistically significant decrease in both ESR and solute clearance across the duration of therapy for all three solutes. We observed decreased membrane performance in 31 (23%) circuits while clinical signs were present in 19 (14.1%). The risk of decreased membrane performance significantly increased over time: 1.8% at T1 (p = 0.16); 7.3% at T2 (p = 0.01); 15.7% at T3 (p = 0.001) and 16.4% at T4 (p < 0.003). Four factors present within 24 h of circuit initiation were independently associated with decreased membrane performance: arterial blood bicarbonate level (OR 1.50; p < 0.001), activated partial thromboplastin time (aPTT; OR = 0.93; p = 0.02), fibrinogen level (OR 6.40; p = 0.03) and Charlson score (OR 0.10; p < 0.01). COVID-19 infection was not associated with increased risk of decreased membrane performance. Regular monitoring of ESR might be appropriate in selected patients undergoing CKRT.

## Introduction

The recent COVID-19 outbreak and associated shortages has brought to the forefront the need for resource optimization. This is particularly true for continuous kidney replacement therapy (CKRT), a common and resource-consuming therapy^1^. Optimization of CKRT has two key objectives: to reach the longest possible circuit lifespan and to maintain high solute clearances throughout the therapy. Improved vascular access, staff training and generalization of regional citrate anticoagulation (RCA) have enabled to reach the first of these objectives. It has become increasingly common to achieve a filter lifespan of 72 h, the maximum duration recommended by most manufacturers. However, prolonged filter lifespan is unlikely to be associated with clinical benefits if associated with decreased clearance. Indeed, circulation of blood for a long period of time can be associated with progressive layering of proteins onto the inner surface of the filter membrane, a process called clogging. Clogging progressively decreases the membrane’s diffusive and convective capacity and leads to inadequate clearance over time. In addition to clogging, other phenomenon, such as subclinical clotting of hollow fibers or dialysate channeling might decrease CKRT membrane performance over time.

Decreased membrane performance is poorly recognized at the bedside as it does not impact filter lifespan and can be associated with the illusion of normality. In diffusion-based modes, it will not trigger alarms and may remain unnoticed for a long time. Decreased filter performance obviously affects the quality of the therapy and in severe forms leads to metabolic disturbances. Recognition of decreased membrane performance should trigger filter replacement in order to optimize therapy. Unfortunately, to date little attention has been paid to this phenomenon in the medical literature and little is known about its incidence and determinants.

Accordingly, we designed a prospective observational study to evaluate the kinetics of small and middle-size molecules clearance during CKRT in continuous veno-venous hemodialysis (CVVHD) mode with RCA. In addition, we aimed to identify predictors of decreased clearance. We hypothesized that overall solute clearance would remain stable over the duration of the therapy but that a proportion of patients would experience a reduction in clearance over time.

## Methods

### Study design and population

This single centre prospective observational study was conducted between September 14th and December 14th 2020 in the adult intensive care unit (ICU) of the Centre Hospitalier Universitaire Vaudois (CHUV), a tertiary teaching hospital located in Lausanne, Switzerland. The three following criteria had to be met in order for patients to be included in the study: age > 18 years, admission to our intensive care unit and receipt of CKRT in CVVHD-RCA mode for either acute or chronic kidney injury for an expected duration of 72 h. Patients for whom consent could not be obtained or who were enrolled in another conflicting trial were excluded.

### CRRT delivery

Therapies were administered with two sets of devices: either Omni^®^ (B Braun Medical, Melsungen, Germany) with polyethersulfone filters 1.6 m^2^ (Omnifilter^®^) or MultiFiltratePRO^®^ (Fresenius Medical Care AG & Co) with 1.8 m^2^ polysulfone Ultraflux^®^ AV1000S or EMiC^®^2 filters. Maximal scheduled running time for all CKRT devices was set at 72 h as recommended by both manufacturers. Therapies were run according to our local CVVHD-RCA protocol and with standard solutions. Dialysate flow rate was set according to patients' dry weight aiming for a delivered effluent rate between 25 and 30 ml/kg/h, in accordance with KDIGO guidelines.

### Data collection

Patients’ characteristics as well as various physiological, biological parameters were recorded at ICU admission, circuit initiation and at 12, 24, 48 and 72 h (T1, T2, T3 and T4) following circuit initiation. For specific biological values, such as C-reactive protein, activated partial thromboplastin time (aPTT), d-dimers, international normalized ratio (INR), thrombocytes, haematocrit and fibrinogen levels, we considered the most abnormal values within 24 h of circuit initiation. Circuit lifespan was recorded and blood and effluent samples were drawn at T1, T2, T3 and T4 in order to measure urea, creatinine and ß2 microglobulin (ß2MG) levels. Effluent/serum ratio (ESR) at each study time point for each molecule (x), respectively (E_x_/S_x_) were computed and solutes clearances were calculated using the following formula^2^:

$${\text{Clearance}}\left( {\text{x}} \right) = {\text{TEV}} \cdot \left( {{\text{E}}_{{\text{x}}} /{\text{S}}_{{\text{x}}} } \right)/{\text{W}} \cdot \left( {{\text{ml}}/{\text{kg}}/{\text{h}}} \right)$$

where TEV corresponds to the total effluent volume over a 24-h period, E_x_ to the solute concentration in the effluent, S_x_ to the solute concentration in the serum and W to the patient's weight. Of note, circuits with a lifetime of < 24 h were excluded from the analyses since only one sample could be retrieved, hindering any evaluation of membrane performance kinetics. The vast majority of these early circuits’ dysfunction were related to vascular access issues.

### Definition of decreased membrane performance

Since urea is a very small molecule with a sieving coefficient close to 1 with modern membranes, its ESR can serve as an indicator of the membrane’s diffusive capacities. A low ESR for urea is highly suggestive of membrane decreased performance^3,4^. To date, no clear threshold warranting filter change has been determined and values ranging from 0.6 to 0.87 have been proposed^4,5^. Therefore, for the purpose of this study, we defined an urea ESR of less than 0.81, equivalent to the 10th percentile of measured values, as a threshold for *decreased membrane performance*.

In addition, we considered *symptomatic decreased membrane performance* any situation combining metabolic alkalosis, hypernatremia and hypercalcemia occurring during the considered therapy. Similar to Khadzyhnov et al.^6^, these metabolic alterations were attributed to decreased membrane performance only if no other possible alternative or confounding diagnosis prevailed and if these anomalies were reversible after CKRT filter replacement.

### Statistical analyses

Data analysis was performed using the STATA 16 software (StataCorp. 2019. Stata Statistical Software: Release 16. College Station, TX: StataCorp LLC). Continuous variables were summarized by the median and the interquartile range (IQR) and by the number and the percentage for categorical variables. The risk for decreased membrane performance during CKRT was assessed using repeated measurement (T1:T4) outcomes: urea, creatinine and ß2 microglobulin. Decreased membrane performance was defined as ESR under 0.81 (coded 1 and 0 if not), 0.50 (coded 1 and 0 if not) and 0.15 (coded 1 and 0 if not) for urea, creatinine and ß2 microglobulin respectively. Mixed-effects logistic regression model was performed to assess the association between each factor and the outcome. The strength of the associations was reported by the OR (Odds-Ratio) and calculated p-value. Potential interactions were tested and significant associated factors at level *P* < 20% were considered in a backward procedure to fit a multivariable model. For urea, a secondary analysis was performed. Decreased membrane performance coded 1 if ESR under 0.81 at any time (T1:T4) and 0 if not. This transversal analysis was performed using univariable and multivariable logistic regression model. Model diagnosis was performed to ensure that the model fits the data well enough and its performance was assessed using the area under the ROC curve (AUC) and standard.

### Ethical disclosures

This study was conducted in accordance with the Declaration of Helsinki^7^, the Human Research Act (HRA)^8^, the Human Research Ordinance (HRO)^9^ and followed the Strengthening the Reporting of Observational Studies in Epidemiology (STROBE) guideline^10^. The study protocol, as well as the consent procedure, were reviewed and approved by the Ethics Committee Vaud (CER-VD 2020-01584). As this study involved patients in an emergency situation, written informed consent was obtained from each patient or their next of kin before enrolment.

## Results

### Patients’ and circuits’ characteristics

During the study period, 58 patients received KRT in our ICU. Of those, 20 were not eligible: nine received KRT for less than 72 h, eight received intermittent KRT and two had heparin as anticoagulation. In addition, three declined consent and one was enrolled in a conflicting study. Hence, 35 patients (150 CKRT circuits) were included in the study, their characteristics are presented in Table 1.

**Table 1:** Patient’ and renal replacement therapy circuit’ characteristics.

Patients (n)	35
Demographics	
Age, years, median [IQR]	68.0 [14]
Gender, male, n (%)	26.0 (74.3)
Body weight, kg, median [IQR]	80.0 [15.5]
SARS-CoV 2 positive, n (%)	16 (45.7)
Charlson score, median [IQR]	4.0 [3.5]
Baseline eGFR MDRD, mL/min/1.72 m^2^, n (%)^a^	
> 60	16 (50)
30–60	7 (21.9)
< 30	9.0 (28.1)
Characteristics at ICU admission	
SAPS II score at ICU admission, median [IQR]	54.0 [21.5]
Mechanical ventilation within first 6 h, yes, n (%)	20 (57.1)
Noradrenaline > 0.1 µg/kg at ICU admission, yes, n (%)	13 (37.1)
Creatinine at ICU admission, µmol/L, median [IQR]	219.0 [176.5]
Circuits per patient, median [IQR]	4.0 [3.5]
Circuits (n)	150
Circuit duration hours, median [IQR]	66.0 [24.3]
Circuits Excluded (lifespan < 24 h), n (%)	15 (10)
Machine type, n (%)	
B.Braun^®^ Omni™	32 (23.7)
Fresenius^®^ Multifiltrate Pro™	103 (76.3)
Filter type	
Emic-2™	11 (8.1)
Omni Filter™	32 (23.7)
Ultraflux™	92 (68.1)
Therapy interruption duration, hours, median (IQR)	3.0 [3.5]
Parameters at circuit initiation	
SOFA score, median, [IQR]	12.0 [4]
CRP (mg/l), median, [IQR]^b^	161.0 [174]
d-dimers (ng/l), median, [IQR]^c^	8192.0 [11,667.8]
Fibrinogen (g/l), median, [IQR]^d^	5.3 [3]
INR, median, [IQR]^e^	1.1 [0.2]
aPTT (sec), median, [IQR]^f^	42.0 [17]
Thrombocytes (G/l), median, [IQR]	159.0 [192]
Hematocrit (%), median, [IQR]	26.0 [5]

aPTT, activated partial thromboplastin time; CRP, C-reactive protein; ICU, intensive care unit; IQR, interquartile range; INR, international normalized ratio; eGFR, estimated glomerular filtration rate; MDRD, modification of diet in renal disease; RRT, renal replacement therapy; SAPS, simplified acute physiology score; SOFA, sequential organ failure assessment score.

^a^Percentages are expressed for patients with retrievable baseline creatinine (missing data = 3/35).

^b^Missing values for 8 circuits.

^c^Missing values for 65 circuits.

^d^Missing values for 39 circuits.

^e^Missing values for 27 circuits.

^f^Missing values for 12 circuits.

15 circuits with a lifetime less than 24 h (mostly due to vascular access issues), together with three for which T1 sample was missing, were excluded from the analysis. The median circuit lifetime of the 135 circuits included was 66 h (IQR 24.3). Their characteristics are shown in Table 1. 135 circuits were patent at T1 and T2, 107 at T3 and 78 at T4 (Table 2). Blood flow was adjusted at least once in 21 (15.5%) circuits, and dialysate flow in 15 (11.1%) circuits as shown in Supplementary Table S1.

**Table 2:** Filter lifespan and number of filters with low urea, creatinine and ß2 microglobulin clearances over time during continuous kidney replacement therapy (CKRT).

CKRT duration	12 h (T1)	24 h (T2)	48 h (T3)	72 h (T4)
Circuits patent, n (%)	135 (100)	135 (100)	107 (100)	78 (100)
Circuits with clinical signs of decreased membrane performance, n (%)	1 (0.7)	8 (6)	12 (11.2)	4 (5)
Circuits with low urea clearance, n (%)	4 (3)	15 (11)	16 (15)	9 (11.5)
Circuits with low creatinine clearance, n (%)	6 (4.5)	13 (9.6)	16 (15)	11 (14.1)
Circuits with low ß2 microglobulin clearance, n (%)	9 (6.7)	10 (7.4)	18 (16.8)	9 (11.5)

Low urea, creatinine and ß2 microglobulin clearances were defined as ESR under 0.81, 0.50 and 0.15 respectively.

CI, confidence interval; CRRT, continuous renal replacement therapy; ESR, effluent over serum ratio; OR, odds ratio.

### Decreased membrane performance

#### Urea ESR and clearance

**Figure 1:**
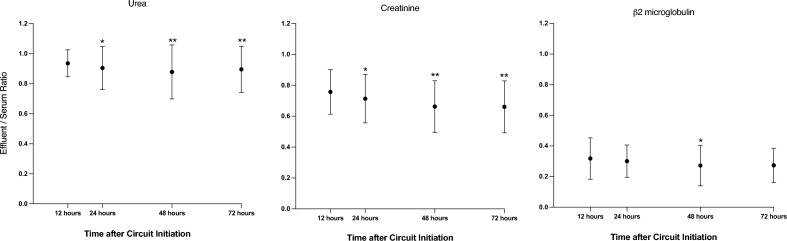
Effluent/serum ratios (ESR) for urea, creatinine and β2 microglobulin over time during continuous kidney replacement therapy (CKRT). ESR for urea, creatinine and β2 microglobulin were compared between timepoints using univariate mixed-effect logistic regression analysis and showed a significant decrease over the course of CRRT. *p < 0.005, **p < 0.001.

**Figure 2:**
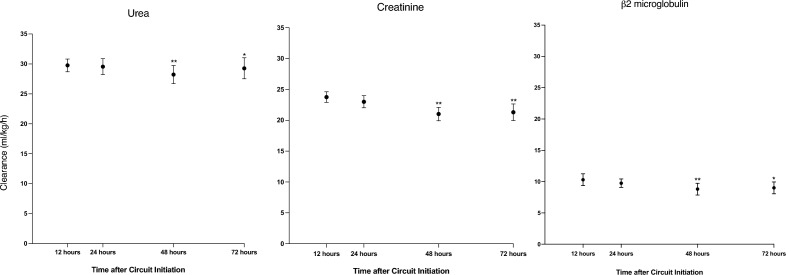
Urea, creatinine and beta-2 microglobulin clearance over time during continuous kidney replacement therapy (CKRT). Clearances for urea, creatinine and β2 microglobulin were compared between timepoints using univariate mixed-effect logistic regression analysis and showed a significant decrease over the course of CRRT. *p value < 0.05, **p value < 0.001.

**Figure 3:**
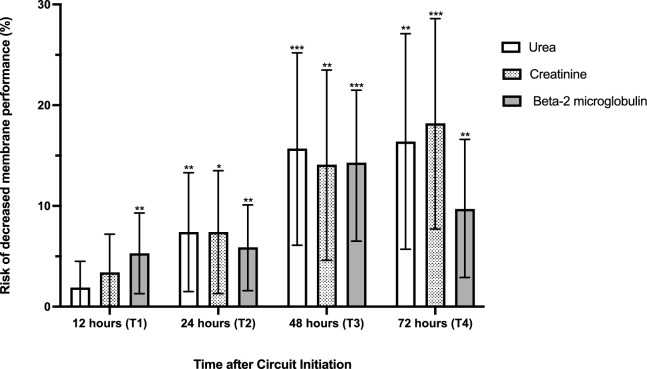
Risk of reduced effluent/serum ratio (ESR) for urea, creatinine and β2 microglobulin clearance over time during continuous kidney replacement therapy (CKRT). The risk of decreased membrane performance was defined as an ESR under 0.81, 0.15 and 0.15 for urea, creatinine and ß2 microglobulin respectively. Univariate mixed-effect logistic regression analysis identified a statistically increased risk of decreased membrane performance over time for urea progressing from 1.8% at T1 (95% Confidence Interval [CI], [0–0.45]; p value = 0.16); 7.3% at T2 ([0.01–0.13]; p value = 0.01); 15.7% at T3 ([0.06–0.25]; p value = 0.001) to 16.4% at T4 ([0.06–0.27]; p value < 0.003). Similar results were reported after univariate mixed-effect logistic regression analysis for creatinine and ß2 microglobulin The risk of decreased membrane performance increased from 3.8% at T1 ([0–0.07]; p value = 0.07); 7.4% at T2 ([0.01–0.14]; p value = 0.02); 14.1% at T3 ([0.05–0.24]; p value = 0.003) to 18.2% at T4 ([0.08–0.29]; p value = 0.001) for creatinine and increasing from 5.3% at T1 ([0.01–0.09]; p value = 0.009), 5.9% at T2 ([0.02–0.1]; p value = 0.006), 14% at T3 ([0.07–0.2]; p value < 0.001) and 9.7% at T4 ([0.03–0.17]; p value = 0.005) for ß2 microglobulin. *p < 0.05; **p < 0.01; ***p < 0.001.

Both ESR and clearance for urea (Figs. 1 and 2), presented minor albeit statistically significant changes across the study duration. Mean ESR at T1, T2, T3 and T4 were 0.94 (SD = 0.09), 0.90 (SD = 0.14), 0.88 (SD = 0.18) and 0.90 (SD = 0.15) respectively. Decreased membrane performance (defined as an ESR for urea < 0.81) was observed in a total of 31 (23%) of the 135 circuits. As shown in Table 2, the proportion of circuits with decreased membrane performance progressively increased over time. Univariate mixed-effect logistic regression analysis identified a statistically increased risk of decreased membrane performance over time progressing from 1.8% at T1 ([95% CI], [0–0.45]; p = 0.16); 7.3% at T2 ([0.01–0.13]; p = 0.01); 15.7% at T3 ([0.06–0.25]; p = 0.001) to 16.4% at T4 ([0.06–0.27]; p < 0.003) with OR at T2 = 7.2 ([1.9–7.5]; p = 0.004), T3 = 25.8 ([5.7–116]; p < 0.001), T4 = 28.2; ([5.5–145]; p < 0.001). These results are shown in Fig. 3. This increased risk of decreased membrane performance over time persisted when multivariate mixed-effect logistic regression analysis was applied with correction for Charlson score, circuit duration, aPTT, arterial bicarbonate level (OR T2 = 11.7 ([2.3–59.5]; p = 0.003), T3 = 77.4 ([10.4–577.5]; p < 0.001), T4 = 118.3; ([12.8–1089]; p < 0.001).

#### Creatinine ESR and clearance

Similar to urea, both ESR and clearances for creatinine presented a minor decrease over time (Figs. 1 and 2), which reached statistical significance at T3 and T4. The mean ESR for creatinine at T1, T2, T3 and T4 were 0.76 (SD = 0.14), 0.71 (SD = 0.16), 0.66 (SD = 0.17) and 0.66 (SD = 0.17) respectively. The proportion of circuits with an ESR for creatinine below the 10th percentile (0.5) increased over time. Univariate mixed-effect logistic regression model analysis demonstrates a statistically increased risk of decreased membrane performance over time: 3.8% at T1 ([0–0.07]; p = 0.07); 7.4% at T2 ([0.01–0.14]; p = 0.02); 14.1% at T3 ([0.05–0.24]; p = 0.003), 18.2% at T4 ([0.08–0.29]; p = 0.001) with OR at T2 = 3.8 ([1.03–14.2], p = 0.04), T3 = 12.5 ([3.08–51.1], p < 0.001), T4 = 21.6 ([4.7–100.3], p < 0.001) as described in Fig. 3.

#### ß2 microglobulin clearance

Again, a minor decrease in both ESR and clearances for β2MG was observed over time (Figs. 1 and 2). The mean ESR at T1, T2, T3 and T4 are 0.32 (SD = 0.14), 0.30 (SD = 0.11), 0.27 (SD = 0.13) and 0.27 (SD = 0.11) respectively. This change was statistically significant at T3 (p = 0.01) for both measures. At T4, statistical significance was reached for ESR and approached for clearance (p = 0.06).

As shown in Fig. 3, the proportion of circuits with an ESR for β2MG below the 10th percentile (0.15) significantly increased over time. The risk of decreased membrane performance assessed using repeated measurement (T1:T4) outcomes with a mixed-effect logistic regression model on a univariate level, progressed from 5.3% at T1 ([0.01–0.09]; p = 0.009), 5.9% at T2 ([0.02–0.1]; p = 0.006), 14% at T3 ([0.07–0.2]; p < 0.001), 9.7% at T4 ([0.03–0.17]; p = 0.005) with OR at T2 = 1.12 ([0.4–2.95], p = 0.8), T3 = 3.26 ([1.3–7.9], p = 0.01), T4 = 2.04 ([0.73–5.7], p = 0.17).

### Independent predictors of decreased membrane performance

As shown in Table 3, multivariable logistic regressions analysis identified in addition to time, four factors independently associated with decreased membrane performance: arterial bicarbonate level at circuit initiation (OR = 1.57 [1.24–1.99], p < 0.001), the aPTT value and fibrinogen level within 24 h of circuit initiation (OR = 0.93 [0.88–0.99], p = 0.025) and (OR = 6.4 [1.2–34], p = 0.028) respectively, and the Charlson score (OR = 0.1 [0.02–0.6], p < 0.01). The resulting model could predict the risk of decreased membrane performance with an AUC of 0.94. COVID-19 and anticoagulation therapy were not associated with decreased membrane performance.

**Table 3:** Predictors of renal replacement decreased membrane performance (transversal analysis).

Independent predictors of decreased membrane performance	Odds ratio	95% confidence interval	p value
Arterial bicarbonate level at circuit initiation	1.57	1.24–1.99	< 0.001
Highest aPTT value within 24 h of circuit initiation	0.93	0.88–0.99	0.025
Highest fibrinogen levels within 24 h of circuit initiation	6.47	1.22–34.2	0.028
Charlson score	0.1	0.02–0.6	0.01

Multivariable logistic regressions analysis identified the four following independent predictors of decreased membrane performance (defined as urea ESR < 0.81): arterial bicarbonate level at circuit initiation, the highest aPTT and fibrinogen values within 24 h of circuit initiation and patient Charlson score. Integration of these four variables into a logistic regression model established a score able to predict the risk of decreased membrane performance with an area under the ROC curve equal to 0.94.

ESR, effluent over serum ratio; ROC, receiver operating characteristic.

### Clinical signs of decreased membrane performance

Metabolic derangements compatible with decreased membrane performance were recorded in 19 (14.0%) circuits corresponding to five (14.3%) patients. In those circuits, a decrease in urea ESR under 0.81 was observed in all cases. The reduction in urea ESR was present 12–24 h prior to the symptoms in 7 (37%) circuits and simultaneously in 12 (63%) circuits. However, low urea ESR was not associated with clinical symptoms in 12 (8%) circuits.

## Discussion

### Key findings

We conducted a single centre prospective observational study including 35 critically ill patients undergoing CKRT in CVVHD-RCA mode. We computed ESR and clearances for small and middle size molecules at different time points in 135 consecutive circuits. We found that the proportion of circuits with low permeability to these molecules increased over time resulting in a small but statistically significant decrease in their performance over time. We identified four independent predictors of decreased membrane performance: arterial bicarbonate level at circuit initiation, aPTT and fibrinogen levels during the first 24 h and the Charlson score. Finally, we observed that decreased ESR was associated with clinical signs of decreased membrane performance and could precede symptoms by 12 to 24 h.

### Comparison with previous studies

A progressive decrease in membrane performance has been reported in CVVH^11^ and CVVHDF^2,4^ modes. This decrease translated into a difference between prescribed and delivered and dose as illustrated by an ancillary analysis of a randomized controlled trial^2^. However, the clinical relevance of decreased membrane performance in CVVHD mode remains largely unknown. An in vitro study with AN69 membranes reported a higher solute clearance over time in CVVHD mode compared with CVVH^12,13^. Indeed, the filtration process might facilitate protein and blood components layering onto the membrane. In a small series, Ricci et al. have reported similar solute clearance between diffusive and convective therapies^14^. In the 15 circuits used in CVVHD mode, clearance for urea and creatinine remained stable across study time points while a significant decrease in β2MG clearance was observed. In this study, heparin anticoagulation was utilized, and filter lifespan was much shorter than in the present study (median 37 h) hence, it might have been underpowered to detect significant changes in urea or creatinine clearances^14^.

In a German series, CVVHD with RCA was associated with stable clearance of numerous small and middle size molecules^15^. However, in this study authors utilized a super-high flux membrane, which might greatly minimize clogging. These results might therefore not be applicable to standard membranes. To the best of our knowledge, our study is the first the report serial ESR and clearances for urea, creatinine and β2 microglobulin using standard membranes (< 10% of super-high flux membranes).

There is limited data on the predictors of decreased membrane performance. In line with our results, Khadzyhnov et al. have observed a higher fibrinogen level in patients with clinical signs of decreased membrane performance compared to those without^6^. Conversely, they also observed a higher incidence of decreased membrane performance in patients with COVID-19, a finding that was not replicated in our data possibly. Of note, evaluation of the decrease in membrane performance in this study was limited to symptomatic patients since no serial measurement of ESR or clearance were performed.

However, the progressive clotting of blood within some of the hollow fibers is also to be taken into consideration as it might decrease the effective surface area leading to a reduction in diffusive solute clearance. This mechanism would explain the observed association in our study between decreased membrane performance, activated partial thromboplastin time and fibrinogen level. Another alternative mechanism would be channeling of dialysate in the hollow fiber bundle whereby as the treatment proceeds dialysate flow becomes less uniform.

### Implication for clinicians and policy makers

The clinical impact of decreased solute clearance during CKRT still needs to be established. Nevertheless, we have observed that a reduction in solute clearance, defined by a urea ESR < 0.81, was highly sensitive to predict metabolic alterations and preceded them by 12 to 24 h in over one-third of cases. The identification of specific factors as predictors of loss of filter efficacy enables to identify patients at particular risk of decreased membrane performance during CKRT. In those patients, sequential evaluation of membrane performance with urea ESR could be considered as regularly as every 12 to 24 h throughout CKRT. These measurements are easy to perform and inexpensive.

### Strengths and limitations

This study has several strengths. First, to the best of our knowledge, it is the first prospective study to report sequential clearances of small and middle molecules throughout CKRT duration in RCA-CVVHD mode with standard membranes and to assess their correlation with clinical complications. Second, we were able to conduct detailed mixed model analyses on univariate and multivariate levels, minimizing confounding effect thereby strengthening the quality of our statistical results.

It is however limited by its monocentric nature and the small sample size, giving rise to only a limited number of observations. The extent of possible statistical comparisons was therefore restricted, hindering us from establishing robust clinical recommendations based on our findings. In addition, we did not record pre and post filters pressure and therefore are unable to assess the clotting component of the observed decrease in membrane clearance. However, the long (66 h) median filter lifespan makes major clotting issues unlikely in our cohort. The observed decrease in membrane performance might have been confounded with decrease in dialysate flow setting by clinicians. However, as shown in Supplementary Table S1, the number of these changes were very low and more likely to correspond to a response to metabolic alterations. Finally, we have not explored other thresholds to define decreased membrane performance. However, our results were consistent with three different molecules of different sizes and appear consistent with published literature.

## Conclusion

We observed a small but statistically significant decrease in solute clearance over time during CKRT in RCA-CVVHD mode. In addition to the influence of time, four independent factors were identified as predictors for decreased membrane performance: blood bicarbonate levels, aPTT, fibrinogen levels and the Charlson score. Regular monitoring of urea ESR could be advised in those patients. Further studies are warranted in order to validate and explore the magnitude of the clinical implications of these findings.

### Supplementary Information

Supplementary Table S1.

## Data Availability

The datasets used and analyzed during the current study are available from the corresponding author on reasonable request.
