# Supplementary material for: Kinetics of small and middle molecule clearance during continuous hemodialysis
Source: Sci Rep. 2023 Aug 9;13:12905. doi: 10.1038/s41598-023-40075-y (PMC10412530; doi:10.1038/s41598-023-40075-y)
Supplement: Supplementary file 1 — Supplementary Table S1. [file 41598_2023_40075_MOESM1_ESM.pdf]

Kinetics of Small and Middle Molecule Clearance during Continuous Hemodialysis

Livia Whiting[a], Nathan Bianchi[a], Mohamed Faouzi[b], Antoine Schneider[a,c]

[a] Service de médecine intensive adulte, Centre Hospitalier Universitaire Vaudois (CHUV), Lausanne

[b] Division of Biostatistics, Center for Primary Care and Public Health (UNISANTE), University of Lausanne Switzerland

[c] Faculty of Biology and Medicine, University of Lausanne, Lausanne Switzerland

Short Title: Kinetics of small and middle molecule clearance during CKRT

Corresponding Author:

Dr Antoine Schneider, MD PhD

Service de médecine intensive adulte (SMIA)

Centre Hospitalier Universitaire Vaudois (CHUV)

Rue du Bugnon 46

1011 Lausanne, Switzerland

E-mail: antoine.schneider@chuv.ch

	12 hours (n=135)				24 hours (n=135)				48 hours (n=107)				72 hours (n=78)			
	Dialysate Flow		Blood Flow		Dialysate Flow		Blood Flow		Dialysate Flow		Blood Flow		Dialysate Flow		Blood Flow	
	Increase	Decrease	Increase	Decrease	Increase	Decrease	Increase	Decrease	Increase	Decrease	Increase	Decrease	Increase	Decrease	Increase	Decrease
All circuits	1 (0.7%)	2 (1.5%)	3 (2.2%)	3 (2.2%)	3 (2.2%)	5 (3.7%)	4 (3%)	5 (3.7%)	1 (0.9%)	1 (0.9%)	3 (2.8%)	0 (0%)	4 (5.1%)	1 (1.3%)	4 (5.1%)	3 (3.8%)
Circuits with decreased clearance	1 (25%)	0 (0%)	1 (25%)	0 (0%)	2 (13.3%)	2 (1.5%)	0 (0%)	1 (0.7%)	1 (6.3%)	1 (6.3%)	1 (6.3%)	0 (0%)	2 (22.2%)	1 (11.1%)	0 (0%)	2 (22.2%)

Table S1. Number of circuits with blood flow and dialysate flow adaptations at 12 hours (T1), 24 hours (T2), 48 hours (T3) and 72 hours (T4) during continuous kidney replacement therapy (CKRT).
